# Measurement of collateral perfusion in acute stroke: a vessel-encoded arterial spin labeling study

**DOI:** 10.1038/s41598-019-44417-7

**Published:** 2019-06-03

**Authors:** Thomas W. Okell, George W. J. Harston, Michael A. Chappell, Fintan Sheerin, James Kennedy, Peter Jezzard

**Affiliations:** 1 Wellcome Centre for Integrative Neuroimaging (FMRIB), Nuffield Department of Clinical Neurosciences, University of Oxford, Oxford, UK; 2 Acute Vascular Imaging Centre, Radcliffe Department of Medicine, University of Oxford, Oxford, UK; 3 Acute Stroke Service, Oxford University Hospitals NHS Foundation Trust, Oxford, UK; 4 IBME, Department of Engineering Sciences, University of Oxford, Oxford, UK; 5 Department of Neuroradiology, Oxford University Hospitals NHS Foundation Trust, Oxford, UK

**Keywords:** Stroke, Magnetic resonance imaging

## Abstract

Collateral perfusion is important for sustaining tissue viability in acute ischemic stroke. Conventional techniques for its visualization are invasive, require contrast agents and demonstrate collateral vessels, rather than measuring perfusion directly. In this study we utilize a non-invasive, non-contrast magnetic resonance imaging (MRI)-based method to directly quantify collateral perfusion in acute stroke patients. Vessel-encoded multi-postlabeling delay arterial spin labeling (ASL) was used to separately quantify the blood flow and blood arrival time from four arteries supplying the brain in patients presenting within 18 hours of stroke onset. Twenty-nine acute ischemic stroke patients were scanned with a median time of onset to first MRI of 3 hours. Collateral perfusion at presentation was associated with tissue fate at 1-week. It sustained tissue prior to reperfusion, but was less effective than direct blood flow at maintaining tissue viability in patients who did not reperfuse. Delay in the blood arrival around the ischemic region was found at presentation and reduced over time but was not consistently associated with collateral perfusion. Vessel-encoded multi-postlabeling delay ASL provides a non-invasive tool for direct measurement of collateral perfusion and delayed blood arrival in acute stroke patients.

## Introduction

Collateral tissue perfusion is an important determinant of tissue outcome in acute stroke^1^, sustaining tissue viability prior to reperfusion, and maintaining blood flow in the longer term^2^. Patients with extensive collateral vessels have better clinical outcomes^2–4^, and collateral vessel status may be used to select those patients who are likely to benefit from recanalization therapies^5–11^. However, measuring collateral perfusion directly to the tissues is challenging and current approaches infer collateral perfusion from the presence of collateral blood vessels or delayed blood arrival^2^.

Arterial spin labeling (ASL) is a non-invasive magnetic resonance imaging (MRI) technique that does not require an exogenous contrast agent. ASL MRI labels the arterial blood in the feeding arteries in the neck using radiofrequency magnetic pulses, and can serially measure absolute cerebral blood flow (CBF) in patients with acute stroke^12–15^. Vessel-encoded pseudocontinuous ASL (VEPCASL)^16^ is capable of mapping perfusion within territories of individual arteries, providing flow information that agrees well with digital subtraction angiography^17^, and does not compromise signal-to-noise ratio or CBF quantification^18^.

The time taken for the labeled blood to reach the tissue, the arterial transit time (ATT), can be calculated from ASL images when data at multiple postlabeling delays (multi-PLD) have been acquired. Delays in blood arrival have been proposed to identify collateral perfusion^2,19–21^, but delayed ATT can also be observed in other settings including vascular disease, ischemia, and in those with microvascular changes^22–25^.

In this study, we present the use of multi-PLD VEPCASL acquired serially in a cohort of patients with acute ischemic stroke. We demonstrate that VEPCASL can concurrently identify collateral perfusion patterns and delayed blood arrival serially in these patients, and assess whether collateral perfusion measured at presentation is associated with tissue fate at follow-up.

## Methods

### Patients

Patients presenting with acute ischemic stroke within 18 hours of symptom onset were recruited and consented under research protocols agreed by the UK National Research Ethics Service Committee South Central – Oxford C (refs: 12/SC/0292 and 13/SC/0362). Inclusion criteria for this analysis were: presenting scan within 18 hours of symptom onset; Diffusion-weighted imaging (DWI) lesion within the middle cerebral artery (MCA) territory; patient or representative able to give a clear medical history and participate in the consent process; age over 18. Patients with a contraindication to MRI, lacunar stroke defined on DWI, or severely impaired conscious level (score greater than 1 on question 1a of the National Institute for Health Stroke Scale) were not enrolled. Serial imaging was performed at presentation, two hours, 24-hours, 1-week, and 1-month, whenever possible. Where thrombolysis was indicated patients underwent the initial MRI scan during the infusion of alteplase if required. No endovascular treatment options were available at the time of the study. All experiments were performed in accordance with the relevant guidelines and regulations and informed consent or agreement from a consultee was obtained from all individual participants included in the study.

### Imaging

All scans were performed on a 3T Verio (Siemens Healthcare, Erlangen, Germany) using a 32-channel head coil. Preliminary scans were as follows: 1) a rapid 3D time-of-flight (TOF) angiogram of the neck (voxel size 0.8 × 0.8 × 1.3 mm, acquisition time 47 s) to position the VEPCASL labeling plane and identify the location of the feeding arteries within this plane: the right and left internal carotid arteries (ICAs), and the right and left vertebral arteries (VAs), as described previously^18^; 2) Diffusion-weighted images (DWI, voxel size 1.8 × 1.8 × 2.0 mm, b = 0 and 1000 s/mm^2^, acquisition time 3 min) to define the ischemic core; and 3) a T1-weighted structural image (voxel size 1.8 × 1.8 × 1.0 mm, acquisition time 4 min) to aid registration.

These were followed by ASL perfusion imaging, using a previously described protocol^14,18^ which builds on the minimum standards outlined in a recent consensus paper^19^. The protocol included a 1.4 s duration VEPCASL pulse train which cycled through eight different vessel-encodings: two non-selective (label and control), two left-right, two anterior-posterior and two diagonal. Two repetitions of these encodings were acquired for each of six nominal PLDs (0.25, 0.5, 0.75, 1.0, 1.25 and 1.5 s) with a repetition time of 4.1 s, giving a total of 96 volumes in 6.5 minutes. Images were acquired with a 2D multi-slice echo-planar imaging readout (voxel size 3.4 × 3.4 × 5 mm, matrix size 64 × 64, 6/8^ths^ partial Fourier, echo time 14 ms, 24 slices acquired in ascending order)^18^. The time to acquire all slices was 1085 ms, meaning the average effective PLDs across the brain were 0.79, 1.04, 1.29, 1.54, 1.79 and 2.04 s. Calibration scans were acquired with both head and body coils for signal reception to allow for correction of coil non-uniformity and quantification of absolute CBF.

Finally, a T2-weighted turbo spin echo fluid attenuated inversion recovery (FLAIR) acquisition (voxel size 1.9 × 1.9 × 2.0 mm, echo time 96 ms, acquisition time 2 min) was performed at the 1-week timepoint to define final infarct^26^.

### Image processing

Images were processed using the FMRIB software library^27^ and Matlab (MathWorks, Natick, MA, USA), as described previously^14,18^. Pre-processing included motion correction of the VEPCASL raw data^28^, brain extraction^29^ and segmentation of the T1-weighted image^30^, and correction of the VEPCASL data for receive coil non-uniformity. Within-timepoint registration across imaging modalities was achieved using linear registration^28^, but across timepoints non-linear registration^31^ was used to account for tissue distortion^26^.

Separation of the signals arising from each brain-feeding artery in the vessel-encoded data was achieved using a Bayesian maximum *a posteriori* solution^32^ to the general framework for vessel-encoded analysis^33^, which can account for some patient movement between the TOF and VEPCASL acquisitions. Image calibration was achieved by non-linearly registering a ventricle mask from standard space via the patient’s T1-weighted image on to the VEPCASL calibration image to allow estimation of the equilibrium magnetization of cerebrospinal fluid, which is then converted into the equilibrium magnetization of blood. The general ASL kinetic model^20^ was fitted to each arterial component separately using a variational Bayes algorithm^34^ to yield CBF and ATT estimates from each feeding artery within each voxel. To simplify further analysis, weighted ATT maps were calculated by multiplying the CBF and ATT maps, summing across all feeding arteries and then dividing by the total CBF in each voxel. In voxels supplied by a single artery, the weighted ATT is therefore equal to the ATT of this dominant arterial component, but in voxels supplied by multiple arteries it represents the weighted average ATT across these arteries.

### Definitions and regions of interest

For the patients in this study with strokes in the MCA territories, Direct CBF was defined as the blood flow to a voxel from the ipsilateral ICA, and Indirect CBF was the sum of the CBF from all arteries other than the ipsilateral ICA. Therefore, for tissue within the MCA territories, which is normally supplied by the ipsilateral ICA, a change in collateral perfusion originating from the contralateral ICA or the VAs should therefore be reflected by a change in the Indirect CBF.

Each patient was scored on the Modified Thrombolysis in Cerebral Infarction (mTICI) scale using the 24-hour CBF maps when available^7^, with patients categorized as reperfusers (mTICI = 2b or 3) or non-reperfusers (mTICI = 0, 1 or 2a)^14^.

The ischemic core at presentation was defined using semi-automated delineation of the apparent diffusion coefficient (ADC) map^26^ below an externally validated threshold^35^ of 620 × 10^−6^ mm^2^/s. Final infarction was defined preferentially on the 1-week FLAIR, or on the 24-hour trace DWI if the 1-week timepoint was not available^26^. These masks enabled two specific ROIs to be generated which were not derived from a perfusion-based definition of tissue at risk:

1. Surviving tissue: The co-registered final infarct mask was dilated using an empirically defined 10 mm radius spherical kernel before subtracting the original mask. This ROI was generated to investigate Indirect CBF and ATT in tissue that was close to the infarct but survived.
2. Peri-core: The ischemic core mask was dilated using a spherical kernel of radius 20 mm before subtracting the original mask. This ROI was generated to assess the relationship between Direct and Indirect CBF at presentation and risk of subsequent infarction. A larger radius than the surviving tissue ROI was chosen to increase the number of voxels selected, particularly surviving voxels.

Equivalent contralateral ROIs were generated in the same fashion from mirrored ischemic core and final infarct masks. All ROIs were restricted to gray matter and manually checked to ensure no voxels from the opposite hemisphere were included. The gray matter mask was derived from the presenting segmented T1-weighted structural image, registered into the space of the ASL data and thresholded at a partial volume of greater than 0.5^14^.

### Analysis

All data sets were included in the analysis unless affected by significant motion artefacts (assessed in a blinded manner by a clinician, GH), no follow-up data were available to define the final infarct or the ROIs did not contain any voxels after transformation to ASL space and gray matter masking.

The ability of VEPCASL to identify the presence and prevalence of collateral perfusion in tissue that survived was quantified by measuring Indirect CBF in the Surviving Tissue ROI as a proportion of the total CBF. Both the proportion of Indirect CBF and the ATT were compared to the contralateral ROI at presentation and 1-month for those patients where both scans were available. Patient level data were also presented using imaging from all patients at all available timepoints, to reduce any bias arising from exclusion of those lost to follow up. Two-way analysis of variance (ANOVA) of both measures were used to compare Surviving Tissue to the contralateral ROI across the timepoints. If these were significant, post-hoc t-tests were also performed to compare the results within each timepoint.

The effect of collateral perfusion, as measured by VEPCASL, on tissue viability was assessed by measuring the relationship between the Indirect CBF for each voxel in the Peri-Core ROI and whether or not it survived. Voxels that received more than 25 ml/100 g/ of Direct CBF were excluded^36–38^, leaving only those with a significant risk of infarction if collateral perfusion were not present. Tissue survival was defined as those voxels that fell outside the final infarct mask. The proportion of surviving voxels was calculated across 10 ml/100 g/min Indirect CBF ranges. Statistical significance was assessed by comparing the proportion of surviving tissue in voxels with Indirect CBF above and below 25 ml/100 g/min using a binomial proportion test. In order to investigate the effect of reperfusion on this relationship, the analysis was repeated after splitting the patients into reperfusion and non-reperfusion subgroups.

To assess whether there was a difference between the effect of Direct and Indirect CBF on tissue fate, and whether this depended on reperfusion status, the proportion of all voxels within the Peri-Core mask that survived was separately quantified across a range of Direct and Indirect CBF values.

## Results

**Figure 1:**
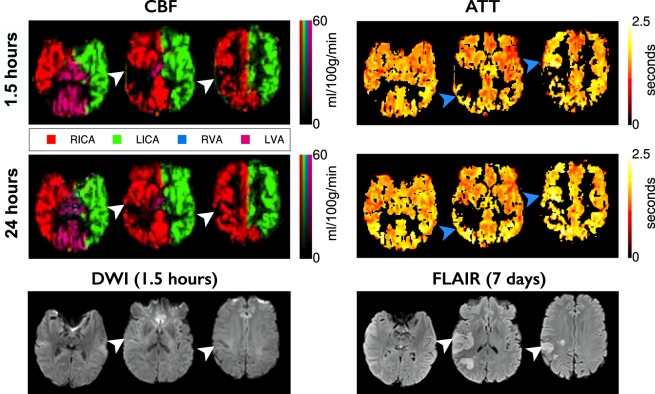
Example data from a patient with no apparent collateral perfusion. CBF and ATT maps from the presenting and 24-hour scans, with the one week T2-weighted FLAIR and presenting DWI, registered to the presenting T1-weighted structural image. The CBF maps are color-coded according to the arterial origin of the blood signal, as shown in the legend. The CBF deficit at presentation in the right MCA territory partially reperfuses by 24-hours, but nevertheless results in infarction (white arrowheads). No collateral CBF to the ischemic area is apparent. In areas around the ischemic region the ATT appears delayed compared to the contralateral hemisphere (blue arrowheads). Times are from symptom onset. ATT values are displayed for voxels with CBF of greater than 25 ml/100 g/min.

**Figure 2:**
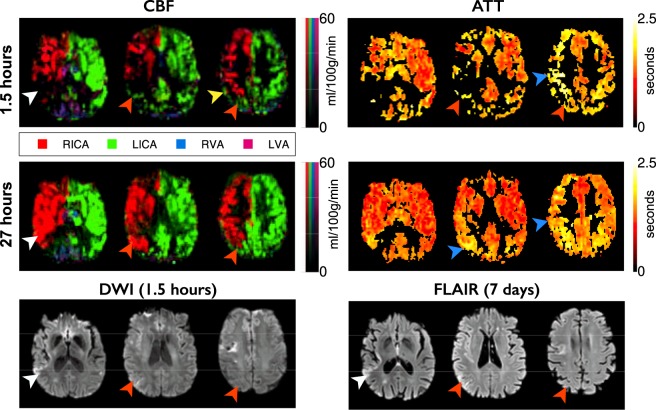
Example data from a patient with inter-hemispheric collateral flow. Images are arranged and labeled as per Fig. 1. The presenting CBF deficit in the right MCA territory reperfuses by 27 hours, but tissue in this region has already infarcted (white arrowheads). Other regions receive collateral perfusion originating from the LICA, probably through the anterior cerebral arteries (orange arrowheads). Some collateral supply appears to flow through pial collaterals (yellow arrowhead). Following reperfusion, the vascular territories revert to a standard configuration, and regions that received collateral perfusion survive. Extended ATT in regions receiving collateral perfusion are shown, but also in regions distal to the ischemic region, which persist even after reperfusion (blue arrowheads).

**Figure 3:**
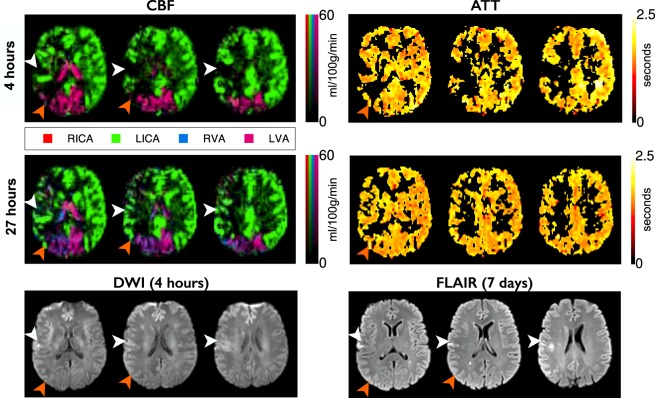
Example data from a patient with RICA occlusion. Images are arranged and labeled as per Fig. 1. RICA occlusion has resulted in the LICA providing blood to both the right anterior cerebral artery and right MCA territories. The small perfusion deficit at presentation leads to infarction despite reperfusion at 27 hours (white arrows). Collateral perfusion from the posterior circulation, which regresses after reperfusion, spares tissue from infarction (orange arrows). Despite the complete collateral supply to the RICA territory there is no apparent delay in ATT.

29 patients were included in this study. Patient demographics are listed in Table 1. The number of multi-PLD VEPCASL scans completed at presentation, two hours, 24-hours, 1-week and 1-month was 24, 17, 20, 17 and 18, respectively. Of these 5, 5, 7, 6 and 0 data sets were excluded at each of the respective timepoints due to the presence of significant motion artefacts. A further 4, 6, 2, 2 and 3 data sets were excluded due to a lack of follow-up infarct mask availability or no voxels within the ROIs, leaving 15, 6, 11, 9 and 15 data sets at each respective time point for analysis. Representative multi-PLD VEPCASL data, clearly showing the phenomena of collateral perfusion and delayed blood arrival at the level of the individual, along with DWI and FLAIR images, can be seen in Figs 1–3.

**Table 1:** Patient demographics.

Mean age (SD), yrs	75.7 (14.6)
Female sex, %	66
Thrombolysed, %	48
Prior stroke/transient ischemic attack, %	38
Hypertension, %	62
Diabetes mellitus, %	14
Atrial fibrillation, %	45
Cigarette smoker (current), %	12
Median NIHSS (IQR)	13 (14)
Median emergency department to MRI (IQR), h:mm	1:27 (0:53)
Median onset to MRI (IQR), h:mm	3:00 (1:54)
Median presenting lesion volume (IQR), ml	10.5 (26.2)
Median final infarct volume (IQR), ml	17.1 (52.2)

NIHSS: National Institute for Health stroke scale; SD: standard deviation; IQR: interquartile range.

### Surviving tissue

**Figure 4:**
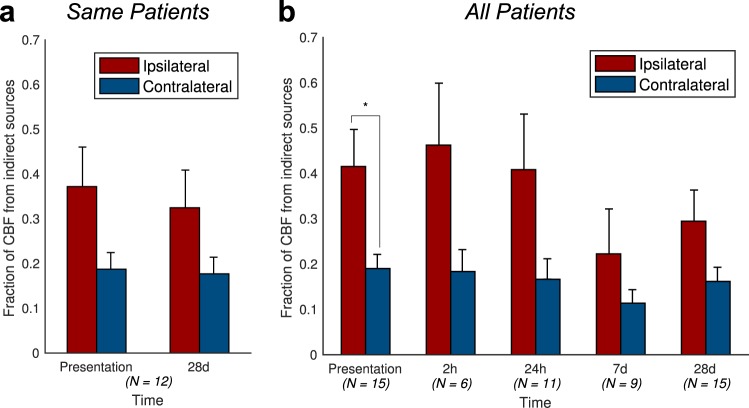
Patient-level collateral perfusion analysis. The fraction of Indirect CBF (blood that does not arise from the ipsilateral ICA), averaged across patients, is much higher within the Surviving Tissue mask than within the contralateral mask, especially at presentation: (**a**) patients with imaging both at presentation and 1-month; (**b**) any available imaging from all patients at each timepoint. The number of patients contributing to the data at each timepoint is quoted below each bar. Error bars represent the standard error. In both cases, the ipsilateral-contralateral difference is significant (ANOVA, p < 0.05). Post-hoc t-test significance (p < 0.05) is marked with an asterisk (*).

For patients with ASL data available at both presentation and 1-month, there was a significantly greater proportion of Indirect CBF in the Surviving Tissue ROI than the contralateral ROI (ANOVA p = 0.016, Fig. 4a), which was most marked at presentation (0.37 vs 0.19), indicating the presence of collateral perfusion. There was no significant effect of timepoint on the proportion of Indirect CBF in Surviving or Contralateral ROIs (p = 0.7). Including all patients at all times demonstrated similar results, with a significant difference between the ipsilateral and contralateral ROIs (ANOVA p = 0.0001, t-test at presentation p = 0.01, Fig. 4b).

**Figure 5:**
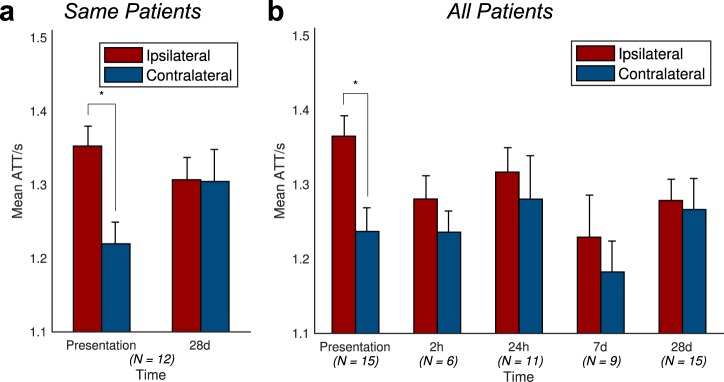
Patient-level arterial transit time (ATT) analysis. The mean ATT of blood within the Surviving Tissue region of interest is higher than that of the contralateral mask at presentation, but this difference decreases with time. (**a**) Patients with imaging both at presentation and 1-month; (**b**) any available imaging from all patients at each timepoint. The number of patients contributing to the data at each timepoint is quoted below each bar. Error bars represent the standard error. In both cases, the ipsilateral-contralateral difference is significant (ANOVA, p < 0.05). Post-hoc t-test significance (p < 0.05) is marked with an asterisk (*).

Delayed blood arrival within the Surviving Tissue ROI was observed relative to the contralateral mask (ANOVA p = 0.047, Fig. 5a), but the delay was only marked at presentation, with the mean ATT values being 1.35 s in Surviving Tissue versus 1.22 s on the contralateral side (t-test p = 0.005, n = 12). There was no significant effect of timepoint on the ATT values (p = 0.6). Including data from all patients at all times yields results showing a similar trend for reduction in the mean ATT difference between Surviving and contralateral ROIs over time, although only the effect of ROI was significant (ANOVA p = 0.047, t-test at presentation p = 0.001, Fig. 5b).

At the level of the individual (Figs 1–3) it was observed that collateral perfusion and delayed blood arrival do not always coincide, with persistent ATT increases still visible after reperfusion in some cases. In Fig. 1, no apparent collateral perfusion is present but the asymmetrical ATT demonstrates that in addition to incomplete reperfusion there are persistent arrival time delays, even in the absence of Indirect CBF. In Fig. 2, inter-hemispherical collateral perfusion is apparent at presentation, but despite the seemingly complete reperfusion and resolution of the collateral flow, ATT delays also persist at 24-hours in the region of the presenting perfusion deficit. In contrast, delayed blood arrival is not evident in Fig. 3 either before or after reperfusion despite blood flow to the right ICA territory originating entirely from the contralateral ICA.

### Peri-core

**Figure 6:**
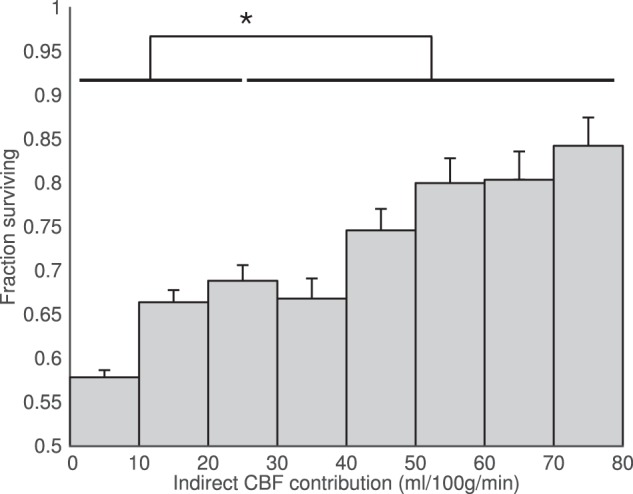
Voxel-level tissue survival as a function of collateral perfusion. In Peri-Core voxels greater Indirect CBF at presentation significantly increases the probability of tissue survival at one week. Each bar represents the fraction of voxels, across all patients, that survive within each given range of Indirect CBF. The asterisk (*) represents the significant difference in survival fraction for tissue receiving less than 25 ml/100 g/min of Indirect CBF compared to that receiving more than this value (p < 0.0001).

Within Peri-core voxels with less than 25 ml/100 g/min Direct CBF at presentation, which were at risk of infarction in the absence of collateral flow, there was a clear increase in tissue survival fraction in voxels with higher levels of Indirect CBF (Fig. 6). This indicates that collateral perfusion, as measured by VEPCASL, has a meaningful impact on tissue fate. The tissue survival fraction in voxels receiving less than 25 ml/100 g/min of Indirect CBF was significantly lower than in those above this threshold (60.3% versus 75.2%, p < 0.0001). The relative increase in tissue survival fraction between low (0–10 ml/100 g/min) and high (70–80 ml/100 g/min) levels of Indirect CBF was 45%.

**Figure 7:**
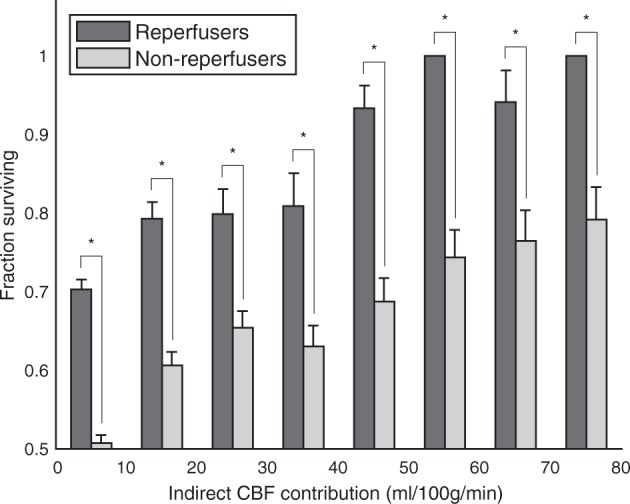
Voxel-level tissue survival analysis within the Peri-Core ROI, as shown in Fig. 6, plotted separately for patients that reperfuse and those that do not. In both groups, increased Indirect CBF, and thus collateral perfusion, at presentation improves the likelihood of tissue survival (p < 0.0001). However, the probability of tissue survival is significantly increased in patients that reperfuse at all indirect CBF ranges, according to binomial proportion tests (*p < 0.05).

A similar relationship between Indirect CBF and tissue survival was seen in both the reperfusion and non-reperfusion subgroups separately (Fig. 7). This association was significant in both cases (p < 0.0001), although as expected, the fraction of tissue surviving was always greater in voxels of patients who reperfused (p < 0.05).

**Figure 8:**
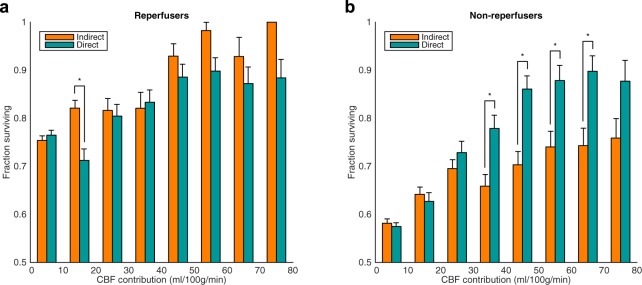
Voxel-level association between the Direct and Indirect CBF contributions and tissue survival. Analysis was performed in all Peri-Core voxels, for patients who reperfused (**a**) and patients who did not reperfuse (**b**) separately. *p < 0.05 using a binomial proportion test.

Across all voxels within the Peri-Core ROI, regardless of Direct CBF value, increases in both Direct and Indirect CBF lead to similar improvements in tissue survival for patients that reperfused (Fig. 8a). In patients who did not reperfuse, greater Direct CBF led to significantly larger tissue survival fractions than equivalent levels of Indirect CBF.

At the level of the individual, the ability of Indirect CBF to sustain tissue viability prior to reperfusion is highlighted in Figs 1–3. No collateral perfusion is seen in Fig. 1, and the hypoperfused tissue has infarcted at follow up. In contrast, in Figs 2 and 3 the CBF at presentation in some regions is maintained from collateral sources. The Indirect CBF supports tissue survival pending reperfusion by 24-hours, where it occurs. Indirect CBF was observed both from contralateral and posterior circulation sources: in Fig. 2, CBF in the right hemisphere was sustained by blood from the left ICA, before reperfusion from the right ICA at 24-hours; in Fig. 3, where both MCA territories are supplied by the left ICA due to a right ICA occlusion, at presentation blood from the vertebral arteries sustains the tissue prior to reperfusion from the left ICA at 24-hours.

## Discussion

In this study we have shown that multi-PLD VEPCASL is capable of directly visualizing collateral perfusion and delayed blood arrival serially in acute stroke patients. Unlike conventional imaging approaches, this technique is non-invasive and capable of directly visualizing collateral perfusion at a tissue level, rather than just identifying collateral vessels. A VEPCASL-derived measure this phenomenon, Indirect CBF, measured at presentation was shown to be associated with tissue fate, both within individual patients and across the cohort. In particular, our results suggest that Indirect CBF at presentation can compensate for a lack of Direct CBF early after acute stroke and prior to reperfusion. However, Indirect CBF was not as effective as Direct CBF in sustaining tissue viability in patients who did not reperfuse. Finally, the multi-PLD protocol used allows delayed blood arrival to be measured, yielding information that is complementary to Indirect CBF in acute stroke.

Indirect CBF accounted for less than 20% of total CBF in the contralateral hemisphere ROI in keeping with chronic cerebrovascular disease^39^. In the Surviving Tissue ROI, Indirect CBF contributed around 40% of the CBF for the first 24-hours after stroke onset. The greater proportion of Indirect CBF in the affected hemisphere was most marked in the short-term, but diminished over time. In keeping with all other perfusion techniques it is impossible to know the arrangement of collateral perfusion before the acute event. However, the temporal changes of the proportion of Indirect CBF within the two hemisphere ROIs is consistent with acute changes in collateral perfusion that have occurred as a result of the stroke, and that regress over time^40^.

As would be expected reperfusion was associated with a greater proportion of tissue survival in the Peri-core ROIs. Indirect CBF increased the chances of tissue survival in the context of reperfusion to a similar extent as Direct CBF. However, in the absence of reperfusion fewer Peri-core voxels survive, and are more likely to do so if they have a greater proportion of Direct rather than Indirect CBF supply. This supports the concept that collateral perfusion is a short-term bridging phenomenon that can sustain tissue viability pending reperfusion of the original feeding vessel in acute stroke^40,41^. At the level of the individual patients, regression of collateral perfusion was observed once reperfusion occurred. Although evidence of collateral perfusion that persisted over several days was noted in one patient, this pattern appeared to be a longstanding, chronic collateralization, rather than the acute sustaining collateral perfusion observed at a group level. The short-term ability of acute collateral perfusion to maintain tissue viability is consistent with data from pre-clinical work^42^, and from clinical trials demonstrating that patients who have more collateral blood vessels have better responses to treatment, even outside conventional time windows^4–6,9–11^.

While the pattern of delayed ATT at presentation was consistent with findings from previous single time-point studies, the serial and individual patient data point to a more complicated relationship between ATT and collateral perfusion^22,24^. Established collateral flow, such as that found in response to an ICA occlusion, can be associated with normal ATT, as shown in Fig. 3. Conversely delayed ATT was present even when collateral flow had regressed (Fig. 2). Although delayed ATT has been shown to associate with collateral blood flow in specific settings, due to the circuitous route blood may take to reach the tissue^21^, it may also result from incomplete recanalization, or increased vascular resistance to flow due to capillary occlusion, edema or endothelial dysfunction^43–45^, and caution is required when interpreting delayed blood arrival as a marker of collateral flow. The use of techniques such as VEPCASL to evaluate changes in both collateral perfusion and blood arrival time simultaneously helps to avoid this ambiguity, and could aid the prediction of short and longer term outcomes, particularly in relation to recanalization therapies.

This study is subject to several limitations. Like all ASL studies the results are limited by voxels that have a very prolonged delay to arrival, by which time the signal has decayed considerably, making CBF quantification very challenging. The inherently low signal-to-noise associated with ASL also means that voxel sizes are a compromise between spatial resolution, signal and acquisition times. This in turn can lead to partial volume contamination of each voxel, although the effect on the results will be reduced when using mean values from large ROIs or when comparing with matched contralateral ROIs.

The use of VEPCASL has allowed the observation of collateral flow between the main brain-feeding arteries in this study, but compensatory flow within the vascular territory of a single artery (e.g. from the right anterior cerebral artery to the right MCA) could not be observed. Labeling a larger number of arterial branches more distally has been demonstrated^46,47^, but this may not be practical in acute patients because of planning time and restricted brain coverage.

The relatively small sample size meant that some of the more subtle trends in the data did not reach significance at the patient level, and we were unable to perform subgroup analyses to assess the impact of comorbidities and current patient medication. The number of data sets available at intermediate time points was also limited by a number of factors, including lack of research scanner availability, changes in the patient’s clinical status, and some patients being transferred to rehabilitation facilities or other clinical units where follow-up scans were not possible. Scans at 28 days were more common since the condition of many patients had improved. However, the loss of data due to motion-related artefact and patient dropout may introduce a bias at a group level, with the exclusion of more severe stroke syndromes. A larger follow-up study would allow collateral perfusion and delayed blood arrival to be studied in greater detail in the future.

The strengths of this study include not using gadolinium-based contrast, meaning serial data could be acquired to track the dynamics of absolute measures of collateral flow, and making it possible to measure both hypo- and hyperperfusion. Vessel-encoding allows absolute measurement of collateral perfusion across large territories in acute stroke. Multiple post labelling delays allows the independent identification of delayed ATT which may give indications of microcirculatory resistance as well as collateral flow, but this would need validation in larger cohorts. Before such an undertaking could occur further work would be required to improve the reliability of this technique, reduce its sensitivity to motion artefact, and validate it against conventional angiography in acute stroke.

## Conclusions

Multi-PLD VEPCASL offers an opportunity to quantify collateral perfusion and delays to blood arrival serially in acute stroke patients and their relationship to tissue survival. Indirect CBF is an important transient determinant of tissue fate following acute stroke, particularly for patients who reperfuse. Delayed arrival time appears to represent more than collateral perfusion and warrants further investigation.

## Data Availability

Summary data that underlie the results presented here are available from the corresponding author upon reasonable request, but individual patient data are not freely available due to the constraints of the consent gained from patients and the restrictions imposed by local regulations at the time of recruitment.
